# Interobserver and intermethod variability in data interpretation of breast strain elastography in suspicious breast lesions

**DOI:** 10.3906/sag-2006-257

**Published:** 2021-04-30

**Authors:** Hale TURNAOĞLU, Kemal Murat HABERAL, Serdar ARSLAN, Meriç YAVUZ ÇOLAK, Funda ULU ÖZTÜRK, Nihal USLU

**Affiliations:** 1 Department of Radiology, Faculty of Medicine, Başkent University, Ankara Turkey; 2 Department of Radiology, Cerrahpaşa Faculty of Medicine, İstanbul University, İstanbul Turkey; 3 Department of Biostatistics, Faculty of Medicine, Başkent University, Ankara Turkey

**Keywords:** Breast, elasticity score, interobserver variability, intermethod variability, strain elastography, strain ratio

## Abstract

**Background/aim:**

Strain elastography has the disadvantage of being operator-dependent. Interobserver variability is observed during image acquisition and interpretation. This study aimed to analyze the interobserver and intermethod variability of strain elastography in image interpretation and evaluate the diagnostic performance combining elasticity score and strain ratio with ultrasonography.

**Materials and methods:**

A retrospective study was conducted on 70 breast lesions evaluated with B-mode ultrasonography and strain elastography. B-mode ultrasonography ﬁndings, elasticity scores, and strain ratio values were evaluated using static images by two radiologists. BI-RADS assessment of the lesions and the decision of both observers as to whether the biopsy was required using B-mode ultrasonography, and the combined ultrasonography+elasticity score, and the combined ultrasonography+elasticity score+strain ratio were compared with the histopathological results. Also, the interobserver agreement was analyzed for all the combinations.

**Results:**

There was very good agreement (weighted κ = 0.865) between the observers for the elasticity scores. Very good agreement was observed between the observers for BI-RADS assessments using the combined ultrasonography+elasticity score and the combined ultrasonography+elasticity score+strain ratio (weighted κ = 0.848, and 0.902, respectively). Area under the curve of B-mode ultrasonography, the combined B-mode ultrasonography+elasticity score, and the combined B-mode ultrasonography+elasticity score+strain ratio, were calculated as 0.859, 0.866, and 0.916 for observer 1, and 0.851, 0.829, and 0.916 for observer 2, respectively. There were no statistically significant differences between the observers’ diagnostic performances in any of the combinations (P = 0.703, 0.067, and 0.972, respectively).

**Conclusion:**

In the evaluation and further assessment of breast lesions, semiquantitative strain ratio calculation may help improve diagnostic accuracy by reducing interpretational variety, when used together with B-mode ultrasonography and elasticity scoring, especially for inexperienced individuals.

## 1. Introduction

In general, malignant lesions are stiffer than normal breast tissue and benign lesions [1]. Real-time strain elastography (SE) is a noninvasive imaging technique that provides information about the stiffness of the lesions [2]. In clinical practice, SE is used as an adjunct technique together with ultrasonography (US) in the further identification of breast lesions [3]. This method may be useful in distinguishing malignant and benign lesions [1] and may reduce the number of unnecessary breast biopsies [4]. There are qualitative and semiquantitative methods in SE. Lesion stiffness can be demonstrated on a color scale for qualitative evaluation, and/or can be expressed as a fat-to-lesion strain ratio for semiquantitative evaluation [5]. In the qualitative method, strain distribution is visualized as a color-coded map that is superimposed on the B-mode image of the conventional US [1]. To standardize the interpretation of this image, Itoh et al. [1] improved the Tsukuba elasticity score. In the semiquantitative method, to obtain the strain ratio, two regions of interest, one in the lesion and one in the adjacent adipose tissue, are used. Typically, the strain ratios of malignant lesions are higher than those of benign lesions [6].

Despite the very good diagnostic performance, SE with freehand compression has the disadvantage of being operator-dependent. The obtained strain image is influenced by the compression technique of the individual performer [7]. Also, this technique is dependent on the organ’s deformability and the operator’s skill, which can critically affect the images and the subsequent interpretation [8,9]. Besides, observers can interpret the same obtained image differently. Therefore, there is interobserver variability during image acquisition and interpretation, which may limit the use of SE in routine clinical practice [10]. In the literature, many studies [8,10,11-18] have been conducted on the reproducibility of SE in obtaining elasticity maps, and inconsistent results have been reported. For interobserver reproducibility, caused by both elasticity image acquisition and elasticity image interpretation, some studies [13,15-17] revealed that the agreement was moderate to good for the elasticity score, while others [8,12] showed significant interobserver performance variability. To overcome this limitation, some studies [19,20] suggested that the semiquantitative evaluation of the lesions with strain ratio could be used as a reliable and constant characteristic regardless of data acquisition or interpretation variability. Little published data on the evaluation of the diagnostic performance of the combined 5-point elasticity scoring and strain ratio methods in the same patients exist. Some studies [19-22] have reported that the strain ratio is highly valuable and more objective than the elasticity score. On the contrary, some others [10,23-25] have concluded that the strain ratio does not improve accuracy. To our knowledge, there is no published data about the effect of the combined 5-point elasticity scoring and strain ratio methods on interobserver variability in SE. This study aimed to analyze the interobserver and intermethod variability of SE in image interpretation and evaluate the diagnostic performance of the combined elasticity score and strain ratio with US. 

## 2. Materials and methods 

### 2.1. Study population

A retrospective study was conducted on 70 breast lesions of 68 patients evaluated with B-mode US and SE, who underwent core or excisional biopsy, at Başkent University Hospital. Institutional review board approval was obtained before the study (KA18/330). Hospital and radiology databases of the last 3 years were evaluated, and 81 patients examined with US and SE were identified. Patients with a history of previous treatments, such as breast surgery, chemotherapy, or radiotherapy, were excluded. In addition to BI-RADS 4 and 5 breast lesions, there were also BI-RADS 3 breast lesions which underwent biopsy based on their clinical evaluation results, such as suspicious palpation findings.

### 2.2. Imaging technique

B-mode US images of the lesions were acquired with a Siemens Acuson S2000 device (Siemens Medical Solutions, Mountain View, CA, USA), using an 18 L6 HD (5.5–18 MHz) linear transducer. To evaluate the stiffness of lesions, SE was performed using a Siemens Acuson S2000 device (Siemens Medical Solutions, Mountain View, CA, USA) with a 9L4 (4–9 MHz) linear transducer. When performing SE, the freehand compression method described by Itoh et al. [1] was used. With the patient in supine position, the US transducer was positioned parallel to the breast lesion. To achieve appropriate contact with the skin, slight pressure was applied through the transducer over the breast tissue, resulting in its displacement by 1-2 mm posteriorly, and coming back to its initial location, and elastography images were acquired. The compression sufﬁciency was adjusted according to quality factor 60 and higher as an adequate value. A region of interest (ROI) box was placed on the targeted lesion, and another ROI box was placed on reference tissue determined to be adjacent fat tissue, to measure strain ratio. Strain ratio was calculated by comparing the strain value of the reference tissue with that of the targeted lesion. 

### 2.3. Data interpretation

B-mode US ﬁndings, elasticity scores and strain ratio values were evaluated by 2 radiologists, retrospectively. For each lesion, 1 or 2 representative B-mode US image/s and 1 elasticity score image recorded in only 1 plane were evaluated. The images were converted into JPEG files and reviewed by the observers during 1 image review session. American College of Radiology’s Breast Imaging Reporting and Data System (BI-RADS) [26] assessments of US, elasticity scores, and final BI-RADS assessments after SE were recorded on a sheet prepared by each observer. One of the radiologists (observer 1) had 8 years of experience in breast imaging and elastography, and the second (observer 2) had 5 years of experience in breast imaging and none in elastography. Radiologists were blinded to each other, and also to patients’ all clinical records, including their ages, complaints, the date of onset of symptoms, mammography findings, and biopsy results. First, lesions were classified by BI-RADS, according to the B-mode US findings. Then, elasticity scores were evaluated, and the lesions were reclassified by BI-RADS according to the combined B-mode US+elasticity score findings. Color maps produced based on the elastography images were assessed using the 5-point elastography scoring system (Tsukuba elasticity score) deﬁned by Itoh et al. [1]. Before the session, the observers reviewed the elasticity scores. According to Tsukuba elasticity score, TS1 and TS2 indicate benign breast lesions, TS3 possible benign lesions, and TS4 and TS5 malignant breast lesions. BI-RADS category of the lesions with elasticity scores of 1, 2, and 3 was either changed to a lower BI-RADS category or was not changed. BI-RADS category of the lesions with elastography scores of 4 and 5 were either changed to a higher BI-RADS category or was not changed. Finally, strain ratio values were seen, and final BI-RADS classification was made according to the combined B-mode US+elasticity score+strain ratio values. For the evaluation of strain ratio values, a cut-off value (2.84) was used [27]. The BI-RADS category of the lesions with a strain ratio value equal to or greater than the cut-off value was either changed to a higher BI-RADS category or was not changed. The BI-RADS category of the lesions with a strain ratio value less than the cut-off value was either changed to a lower BI-RADS category or was not changed. At the end, BI-RADS assessment of the lesions, the decision of both observers on whether the biopsy was required using B-mode US, the combined B-mod US+elasticity score, and the combined B-mod US+elasticity score+strain ratio values were compared with the histopathological results. Also, the interobserver agreement was analyzed for all combinations. 

### 2.4. Statistical analysis

Statistical analyses were performed using the Statistical Package for the Social Sciences (SPSS) software, version 25.0 (IBM Corp., Armonk, NY, USA). According to the results of B-mode US and the combined methods, sensitivity, specificity, positive predictive value (PPV), negative predictive value (NPV), and accuracy of benign/malignant differentiation of masses were analyzed. Receiver operating characteristic analyses were performed to calculate the area under the receiver operating characteristic curve (AUC) for overall performance. Also, for each evaluation, interobserver variability was analyzed with interrater agreement kappa statistics. A kappa value of 0 corresponds to no agreement, a kappa value of 1.0 corresponds to perfect agreement, and a kappa value less than 0 corresponds to disagreement. Kappa values less than or equal to 0.20 indicate poor agreement, values between 0.21 - 0.40 indicate fair agreement, values between 0.41 - 0.60 indicate moderate agreement, values between 0.61 - 0.80 indicate good agreement, and values between 0.81 - 0.99 indicate very good agreement. All non-binary variables were also tested using the weighted kappa statistic. For the pairwise comparison of ROC curves, Medcalc® programme was used. A P-value of <0.001 was considered statistically signiﬁcant [28]. 

## 3. Results

### 3.1. Pathological diagnoses

After histopathological examination, 36 of 70 breast lesions were diagnosed as malignant, and 34 lesions were diagnosed as benign. Pathological diagnoses are demonstrated in Table 1. 

**Table 1:** Pathological diagnoses of the 70 breast lesions.

Malignant lesions	N	Benign lesions	N
Invasive carcinoma	35	Fibroadenoma	16
Ductal carcinoma in situ	1	Sclerosing adenosis	7
		Granulomatous inflammation	3
		Intraductal papilloma	3
		Fat necrosis	2
		Fibrocystic changes	2
		Atypical lobular hyperplasia	1

### 3.2. Interobserver variability for BI-RADS US assessment and the decision with B-mode US findings 

Good agreement (weighted κ = 0.784, P = 0.042) was seen among the observers in terms of BI-RADS assessment of the US. Very good agreement was observed (κ = 0.839, P = 0.000) among the observers in terms of the decision of whether biopsy was required for the lesions. 

### 3.3. Interobserver variability for elasticity scores and the decision with the combined B-mode US findings+elasticity scores; and intermethod variability

Very good agreement (weighted κ = 0.865, P = 0.039) was seen among the observers in terms of elasticity scores. Also, very good agreement was observed (weighted κ = 0.848, P = 0.034) among the observers in terms of BI-RADS assessment with the combined B-mode US+elasticity score. There was very good agreement (κ = 0.822, P = 0.000) among the observers in terms of the decision of whether biopsy was required for the lesions. 

When the observers’ decision of whether biopsy was required using B-mode US assessment alone was compared to using the combined B-mode US+elasticity score, observer 1 had good agreement (κ = 0.696, P = 0.000) while observer 2 had moderate agreement (κ = 0.548, P = 0.000). 

### 3.4. Interobserver variability for the decision with the combined B-mode US findings+elasticity scores+strain ratio values and intermethod variability

Very good agreement was observed between the observers for BI-RADS assessment with the combined B-mode US+elasticity score+strain ratio (weighted kappa = 0.902, P = 0.027). Good agreement was observed between the observers for the decision of whether biopsy of the lesions was required (κ = 0.776, P = 0.000). 

When the observers’ decision of whether biopsy was required using the combined B-mode US+elasticity score was compared to using the combined B-mode US+elasticity scores+strain ratio, observer 1 had good agreement (κ = 0.644, P = 0.000), and observer 2 had moderate agreement (κ = 0.548, P = 0.000).

### 3.5. Diagnostic performances

Diagnostic indexes for B-mode US, the combined B-mode US+elasticity score, and the combined B-mode US+elasticity score+strain ratio are summarized in Table 2. ROC curves for both observers are demonstrated in Figure. 

**Table 2:** Diagnostic indices of B-mode ultrasonography, the combined B-mode ultrasonography+elasticity score, and the combined B-mode ultrasonography+elasticity score+strain ratio assessments.

	OBSERVER 1			OBSERVER 2		
	B-MOD*	B-MOD+ES#	B-MOD+ES +SR¥	B-MOD	B-MOD+ES#	B-MOD+ES +SR¥
Sensitivity (%)	94.4	97.22	97.22	94.44	94.44	97.22
Specificity (%)	35.29	41.18	50	47.06	32.35	50
Positive Predictive Value (%)	60.71	63.64	67.31	65.38	59.65	67.31
Negative Predictive Value (%)	85.71	93.33	94.44	88.89	84.62	94.44
Accuracy (%)	65.71	70	74.29	71.43	64.29	74.29
Area Under the Curve Value	0.859	0.866	0.916	0.851	0.829	0.916

**Figure 1:**
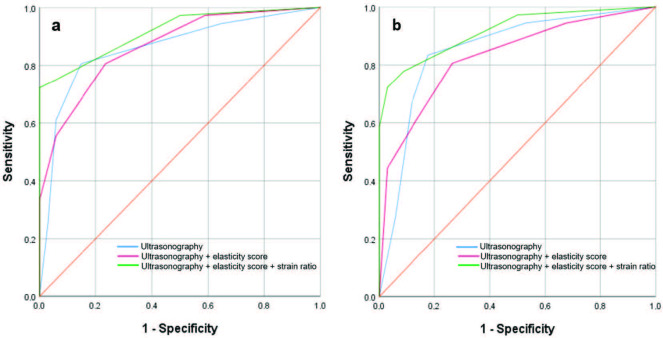
Receiver operating characteristic curves for B-mode ultrasonography, the combined B-mode ultrasonography+elasticity score, and the combined B-mode ultrasonography+elasticity score+strain ratio. a. Observer 1 (area under the curve [AUC] values 0.859, 0.866, and 0.916, respectively) b. Observer 2 (AUC values, 0.851, 0.829, and 0.917, respectively).

### 3.6. Comparison of the ROC curves of observers

There was no statistically significant difference between the observers in terms of their diagnostic performances in any of the combinations. The P-values of B-mode US, the combined B-mode US+elasticity score, and the combined B-mode US+elasticity score+strain ratio assessments were found to be 0.703, 0.067, and 0.972, respectively.

## 4. Discussion

Our study has shown that the use of B-mode US+elasticity score+strain ratio evaluation method increased all diagnostic indices in differentiating benign from malignant breast lesions, in both the experienced and inexperienced observer (Table 2). In the method of the combined B-mode US+elasticity score, for the inexperienced observer, sensitivity did not change but other diagnostic indices were downgraded. All values improved and became equal to that of the experienced observer after the evaluation of the combined B-mode US+elasticity score+strain ratio. Despite the change in diagnostic indices, there was no statistically significant difference between experienced and inexperienced observers in terms of their diagnostic performances in any of the combinations. Even so, the P-value of the combined B-mode US+elasticity score was 0.067, which was lower than the others. Although the difference is not statistically significant, its clinical significance may be discussed at this point. Similar to our study, there are other recent studies [10,29,30] reporting how SE improved the overall diagnostic value of ultrasound although the change in AUC values was not significant. 

Observer variability for SE is an important limitation which may delimitate the use of SE in breast [10,31]. Various factors known to affect elastography images include patient factors such as breast size and density, lesion factors such as size, location, and depth, acquisition process factors, such as the type of US elastography device, the extent of tissue compression, and interpretation variability [1,17]. Performance-related variability in SE may be more significant than inaccurate interpretation [10]. The standardization of image acquisition procedures is the essential point in elastography evaluation. However, even with the same elastographic image, variable interpretations are possible among observers [1]. We reviewed the static elastography images to assess interobserver and intermethod variability according to the interpretational differences in identical elastographic images between experienced and inexperienced observers. We found good-to-very good agreement in elasticity scores evaluation, in BI-RADS assessments of the lesions, and final decisions of the observers, with both the combined B-mode US+elasticity score and the combined B-mode US+elasticity score+strain ratio. Many of the previous studies have described interobserver variability as a limitation of SE and have reported varying levels of agreement. Yoon et al. [10] reported that although agreement for real-time elastography images was fair, moderate-to-good agreement was observed in review of the static elastography images. Similarly, Dong et al. [32] analyzed the observer reproducibility of SE in elasticity image acquisition and elasticity image interpretation. They only evaluated the elasticity scoring and found moderate agreement in elasticity image acquisition process (kappa value: 0.438), and poor agreement in image interpretation process (kappa value: 0.365). Additionally, they reported that despite the signiﬁcant variability, there was no signiﬁcant diﬀerence between the 2 performers in terms of diagnostic performance. Various other studies [13-18,33] which evaluated the elasticity scores alone reported higher kappa values than the aforementioned studies, and the corresponding kappa values ranging from 0.408 to 0.779 were compatible with moderate to good agreement. It has been reported that a 1-h didactic session before US BI-RADS classification improved interobserver agreement [34]. Also, Schwab et al. [33]. stated the importance of training in the interpretation and characterization of breast lesions, and they attributed the good interobserver agreement in their study to 1-week elastography training. Tsukuba score reviewing before the session may also explain the higher kappa values in our study. On the other hand, it is noteworthy that diﬀerent studies used diﬀerent US equipment system. Siemens US system was used in our study, while Hitachi, Siemens, and Philips US systems were used in other studies. US systems may be one of the sources of variation. The scoring system based on Hitachi US system may not exactly match the diﬀerent strain image formation algorithms used [29] and the elasticity images of diﬀerent US systems.

The strain ratio, as a semiquantitative measurement, should provide more objective results. Although the strain ratio is more objective than elasticity scoring because of the display of calculated numeric values on the ultrasound machine, the reported cut-off points of strain ratios varied in the studies. Several studies have found that the strain ratio can determine whether a lesion is benign or malignant using the cut-off points 2.45 [22], 2.84 [27], 3.5 [35], 4.8 [36], and 5.6 [21]. The differences in the cut-off points of the strain ratio have been reported according to the selection of the reference ROI, such as the superﬁcial fat adjacent to the skin layer or the fat tissue at a depth similar to or close to the target mass [4, 19]. Also, the ROI should only contain fat in the fat measurement. However, this may not be possible in the clinical practice every time. On the other hand, improper precompression, especially when the diagnosis was made by a radiologist with inadequate clinical experience, can change the strain ratio [35]. Apart from this, the strain ratio depends on and changes with the study population and the specific elastography machine used [5]. Thus, we used a cut-off value of 2.84 (with a sensitivity of 78.9, and a specificity of 90.7) based on the results of another study which used the same equipment system and was performed in our department [27]. 

In the literature, there are controversial findings on the contribution of the strain ratio assessment to differentiating benign from malignant breast lesions. Zhao et al. [20] found that adding strain ratio to B-mode US BI-RADS analysis of breast lesions improved the specificity of assessment without a loss of sensitivity and concluded that it should be integrated into the daily practice. Zhi et al. [19] stated that the diagnostic performance of strain ratio analysis was better than that of the 5-point scoring system, and that strain ratio analysis can provide a more reliable diagnostic tool in comparison to a 5-point elasticity scoring system. Thomas et al. [22] concluded that the strain ratio measurement can contribute to the standardization of elastography while providing high specificity and sensitivity. Alhabshi el al. [21] found that semiquantitative methods had a significantly higher sensitivity and specificity compared to qualitative strain pattern, and that the combined technique with qualitative and semiquantitative methods can improve the specificity and positive predictive value of breast lesions in the differentiation of benign and malignant lesions. On the contrary, Yerli et al. [37] concluded that elasticity scoring and strain ratio methods combined with B-mode US seem to have a similar diagnostic potential for differentiating between benign and malignant breast masses, and qualitative 5-point scoring is a complementary and sufficient method that increases specificity. However, in their prospective study, in the qualitative evaluation, they used a 5-point scoring method proposed by an Italian multicenter study for lesion classification. In their prospective study, Yoon et al. [10] found that strain ratio did not make any significant improvements in the diagnostic performance or interobserver agreement among 3 performers. They found higher AUC values for strain ratio compared to elasticity scores, but the difference was not statistically significant. Thus, they concluded that it did not offer any additional information, other than the elasticity score. Similarly, in some other studies [5,23-25], no significant difference was observed between qualitative and quantitative assessments. Bojanik et al. [35] found that the combined B-mode US+elasticity scoring had better specificity and accuracy than combined B-mode US+strain ratio in distinguishing benign from malignant breast lesions. However, the best diagnostic performance was achieved when B-mode US was combined with both elasticity score and strain ratio with the area under the curve of 0.973, according to the ROC analysis, and it was found that elastography combined with B-mode US improved the specificity, accuracy, and the positive predictive value. It was concluded that the routine use of such a diagnostic algorithm could reduce the number of unnecessary biopsies. In our study, although there was no statistically significant difference between the combinations in terms of AUC values, we found the best AUC value when the combined B-mode US+elasticity score+strain ratio was used, as in the Bojanik study. However, in our study, while there was a decrease in the AUC value of the inexperienced observer when the combined B-mode US+elasticity score was used, the AUC values of both observers were the same when the combined B-mode US+elasticity score+strain ratio was used. These findings suggest that the elasticity score interpretation is more subjective and may be misleading, especially for inexperienced individuals. Also, these suggest that there is a learning process for the interpretation of elasticity scoring. Semiquantitatively calculated strain ratio, which is more objective, may be helpful in improving diagnostic accuracy by reducing interpretational variety, when used together with B-mode US and elasticity scoring, especially by inexperienced radiologists. However, when obtaining strain ratio, one must keep in mind that there is also a learning process, which influences its reproducibility. The accurately obtained strain ratio value may reduce interpretation differences, bringing even the inexperienced radiologist closer to the accurate diagnosis.

This study has some limitations. It was designed as a retrospective study and was conducted at a single institution. Therefore, only the variability of image interpretation was evaluated. Similar to most of other reports on interobserver variability, our study is based on static images. During real-time examinations, the performers can consider many clinical factors, which may affect the final assessment for some performers and interobserver variability. This was also used by Yoon et al. [10] to explain the relatively low interobserver variability in their study compared with previous reports. Secondly, the acquisition of elasticity scores and the measurement of strain ratio values were performed by more than 1 radiologist. Despite the quality factor, which adjusted compression sufﬁciency, the compression technique carried out by different performers may inﬂuence elastographic images. Moreover, interobserver agreement in image acquisition and the reproducibility of the elasticity scores and the strain ratio were not evaluated in this study. Finally, only the suspicious breast lesions which required histopathological evaluation were included in this study. Thus, the study population was relatively small, and our results are not representative of the complete histological spectrum of breast lesions. Larger prospective studies should be performed to further confirm our results. 

In conclusion, we found good-to-very good agreement in elasticity score evaluation, in BI-RADS assessments of the lesions, and in final decisions of the observers, when the combined B-mode US+elasticity score and the combined B-mode US+elasticity score+strain ratio were used. We also showed that despite there was no signiﬁcant difference in terms of their diagnostic performance, the combined B-mode US+elasticity score+strain ratio evaluation method upgrades diagnostic indices in differentiating between benign and malignant breast lesions, for both the experienced and inexperienced observer. In the evaluation and further assessment of breast lesions, semiquantitatively calculated strain ratio may help improve diagnostic accuracy by reducing interpretational variety when used together with B-mode US and elasticity scoring, especially by inexperienced individuals. 
